# Construct labeling: public trust and scientific credibility

**DOI:** 10.3389/fpsyg.2015.00808

**Published:** 2015-06-09

**Authors:** Arthur G. Bedeian

**Affiliations:** Rucks Department of Management, Louisiana State UniversityBaton Rouge, LA, USA

**Keywords:** construct labeling, wage distributions, wage differentials, CEO/chairman pay

“Constructs are the central means we have for connecting the operations used in a [study] to pertinent theory… [and] mislabelings often have serious implications for either theory or practice.”

—Shadish et al. (2002, p. 65, 71)

Analyses should logically ensue from the data on which they are based (Morely et al., 2001). When such data are mislabeled (whether knowingly or not) and, in turn, research findings are misconstrued, the integrity of a discipline's knowledge base is compromised and scientific progress may be delayed, as future investigations in a topic area are misdirected and resources are wasted pursuing false leads (Stroebe et al., 2012). Moreover, as has become increasingly evident, publicity surrounding such mislabeling, and the ensuing findings, has invariably eroded public trust in science (Ioannidis, 2012). Construct labeling within the scientific community is, thus, a serious matter that requires action.

One among many examples of construct mislabeling is a recent paper, “How Much (More) Should CEOs Make? A Universal Desire for More Equal Pay,” Kiatpongsan and Norton (2014) claim to have compared people's estimates of *chief executive officer* and *unskilled worker actual* wages to their *ideal* for what those wages should be^1^. Kiatpongsan and Norton base their *actual* and *ideal* wage comparisons on four items taken from the 2009 *International Social Survey Programme: Social Inequality IV* (ISSP Research Group, 2012). The ISSP Social Inequality module was administered to 55,659 respondents from 41 countries between February 2009 and November 2010. A review focusing specifically on the data collected in the United States, however, reveals that Kiatpongrsan and Norton's wage-comparison analysis rests on a mislabeling of its inherent core constructs: *viz.*, people's estimates of the wage disparities between what CEOs and unskilled workers are *actually* paid vis-à-vis people's *ideal* wage differential.

## Data

Kiatpongsan and Norton base their *actual* and *ideal* wage comparisons on four items taken from the US version of the ISSP Social Inequality survey. The items, which are not reproduced in Kiatpongsan and Norton's paper, read: (i) “How much do you think a *chairman* of a large national corporation earns?” (emphasis added) (ii) “How much do you think an *unskilled worker in a factory* earns?” (emphasis added) and (iii) “How much do you think a *chairman* of a large national company should earn?” (emphasis added) and (iv) How much do you think an *unskilled worker in a factory* should earn?” (emphasis added). Survey respondents were advised that “[m]any people are not exactly sure about this, but your best guess will be close enough.”

## Methodology

To compare the respondents' actual and ideal estimates of chief executive officers' and unskilled workers' wages, Kiatpongsan and Norton calculated two pay ratios. They computed an estimated actual *CEO-to-unskilled worker* pay ratio was by dividing the respondents' estimated actual *chairmen* pay (Item i) by their estimated actual *unskilled factory worker* pay (Item ii). Similarly, Kiatpongsan and Norton divided the respondents' estimated ideal *chairmen* pay (Item iii) by their estimated ideal *unskilled factory worker* pay (Item iv) to create an estimated ideal *CEO-to-unskilled worker* pay ratio. In generating both ratios, Kiatpongsan and Norton used median rather than mean values because the respondents' actual and ideal earnings estimates were non-normally distributed. In such situations, the median is more representative of an underlying data distribution's center or, in the present instance, the point about which the respondents' actual and ideal estimates tended to cluster.

## Results

In presenting the results of their wage comparisons, Kiatpongsan and Norton reported that American respondents' estimated actual ratio of *CEO* to *unskilled worker* pay (30:1) exceeded their estimated ideal ratio (7:1). They then “compared the estimated pay ratio of *CEOs* to *unskilled workers* with the actual pay ratio for the 16 countries where data on actual pay ratios are available” (emphasis added; p. 589). Relying on data from the AFL-CIO's (2013) Executive Paywatch website, which Kiatpongsan and Norton state shows that, in 2012, “the ratio of the pay of an *average chief executive officer (CEO)* to an *average employee* in the United States” was 354:1 (emphasis added; p. 587), Kiatpongsan and Norton concluded that American respondents “drastically underestimated the gap in actual incomes between *CEOs* and *unskilled workers*” (emphasis added; p. 589), as their estimated actual ratio (30:1) “far exceeded” their estimated ideal ratio (7:1; p. 587).

## Construct labeling

A review of the data on which Kiatpongsan and Norton's analysis rests, however, casts doubt on their findings. Most obviously, the four ISSP survey items that formed the basis for Kiatpongsan and Norton's “estimated” and “ideal” pay ratios asked respondents what they thought a *Chairman of a large national company/corporation* and an *unskilled factory worker* should earn and *not* what they thought an *average CEO* or an *average employee* should earn. Although in some instances the same person may serve as both a company's chairman and CEO, the jobs are not the same. Indeed, in the United States, some 47 percent of S&P 500 companies operate with separate chairmen and CEOs (Spencer Stuart Inc, 2014, p. 23). Similarly, Kiatpongsan and Norton's use of the terms “average employee” and “unskilled factory worker” as if they are synonymous is puzzling. Discussing pay ratios in the United States, the AFL-CIO's (2013) Executive Paywatch website uses neither term, preferring the broader trade-union designation “rank-and-file worker,” a more encompassing classification than *unskilled worker in a factory*, as used in ISSP Items (ii) and (iv) above.

Further germane to Kiatpongsan and Norton's conflating of the chairman and CEO roles is the large difference in compensation typically paid executives who hold the combined positions of CEO and chairman and those who serve as a chairman only. In 2012, for the 180 North American companies each with market capitalization of $20 billion or more, non-CEO chairmen earned a median $492,259 annually as compared with $16,079,480 for CEOs who also served as chairmen (Hodgson and Ruel, 2012). Given this contrast in earnings, and the clear wording of ISSP Items (i) and (iii), Kiatpongsan and Norton's decision to set aside the established distinction between a CEO and a chairman is a mislabeling that, as the opening Shadish et al. (2002) epigram has theoretical and practical implications.

At a deeper level, Kiatpongsan and Norton's use of the AFL-CIO's (2013) Executive Paywatch data on the ratio of CEO to worker pay in the United States involves a further instance of mislabeling. The Executive Paywatch website clearly states that this ratio is “based on [an] AFL-CIO analysis of average CEO pay at 327 companies in the S&P 500 Index, which disclosed 2012 CEO pay data as of April 1, 2013.” Simply put, the ratio does not represent the pay differential between an *average CEO* and an *average employee* in the United States as Kiatpongsan and Norton state. Moreover, this ratio was calculated not by comparing, in the words of Kiatpongsan and Norton, the “incomes of CEO[s] to *unskilled* workers in the United States” (p. 592; emphasis added), but by contrasting the reported average pay of the 327 S&P CEOs for whom data were collected (viz., $12,259,894 annually) with what the AFL-CIO characterized as the “average wages of rank-and-file U. S. workers in 2012” (viz., $34,645 annually)^2^. Thus, in effect, the 354:1 pay ratio Kiatpongsan and Norton use in their analysis is actually a comparison of the average annual pay received by a convenience sample of S&P CEOs with the average yearly earnings of *all* rank-and-file U. S. workers for 2012, and not, as they contend, the “incomes of CEO[s] to *unskilled* workers [factory or otherwise] in the United States.”

## Summary

A study's conclusions may be open to alternative interpretations, but the constructs on which the interpretations are based must be faithfully represented. This is especially true in instances where researchers, such as Kiatpongsan and Norton (2014), intend for their findings to guide “policymakers seeking to understand lay attitudes toward income inequality” (p. 592). Viewed more widely, if as a scientific community, we seek to intensify the dialog between academics and policymakers and to promote the application of rigorous empirical research to public and private sector policy and practice, the manner in which constructs in a study are connected to theory is essential for maintaining our discipline's scientific credibility. Maintaining public trust is likewise essential for securing confidence among the broader population in our capacity to recommend solutions to unresolved social challenges. Simply stated, if we wish to influence policymakers and the lay-public to use our findings, our analyses should logically represent the data on which they are based.

### Conflict of interest statement

The author declares that the research was conducted in the absence of any commercial or financial relationships that could be construed as a potential conflict of interest.

## Footnotes

^1^This example was not selected to necessarily single out Kiatpongsan and Norton (2014) individually, but was selected given its recency and the public-policy importance income equality has already taken in the early weeks in the run-up to the 2016 United States Presidential elections (see, e.g.,: The Hillary pay ratio, 2015, April 15). Other earlier examples of the same mislabeling discussed in the present paper, see: Andersen and Yaish (2012, pp. 16–17), Jasso and Meyersson Milgrom (2008, p. 127) and Osberg and Smeeding (2006, Table 5, Footnote e, p. 465). Other more general examples of mislabeling involve the functional distinctiveness of the dimensions underlying burnout and engagement (Cole et al., 2012) and the empirical redundancy of job satisfaction and organizational commitment (Le et al., 2010). An anonymous referee has also suggested that, beyond construct labeling, scientific progress within a discipline is also hampered by problems in construct development. For more on this latter point, see: Shadish et al. (2002).

^2^Given the information provided, I was unable to locate the Bureau of Labor Statistics table the AFL-CIO's Executive Paywatch website footnotes as the source for the $34,645 figure given as the “average wages of rank-and-file U. S. workers.” This figure, however, appears to have been calculated by multiplying $666.25 (the BLS's seasonally adjusted, preliminary estimate of the “average hourly and weekly earnings of production and nonsupervisory employees on private nonfarm payrolls by industry sector, seasonally adjusted” for July, 2012) times 52 salary weeks or $666.25 52 = $34,645 (Bureau of Labor Statistics, 2012).
